# Supplementary material for: Large-scale genome-wide interaction analyses on multiple cardiometabolic risk factors to identify age-specific genetic risk factors
Source: GeroScience. 2024 Sep 25;47(3):3597–609. doi: 10.1007/s11357-024-01348-0 (PMC12181146; doi:10.1007/s11357-024-01348-0)
Supplement: Supplementary file 1 — Supplementary file1 (DOCX 53870 KB) [file 11357_2024_1348_MOESM1_ESM.docx]

**Supplementary materials**

**Large-scale genome-wide interaction analyses on multiple cardiometabolic risk factors to identify age-specific genetic risk factors**

Linjun Ao, Diana van Heemst, Jiao Luo, Maris Teder-Laving, Reedik Mägi,

Ruth Frikke-Schmidt, Ko Willems van Dijk, Raymond Noordam

Contact information: Linjun Ao

Department of Human Genetics, Leiden University Medical Center, Albinusdreef 2, 2333 ZA Leiden, the Netherlands

Tel: +31-071-52-69475

Email: [l.ao@lumc.nl](mailto:l.ao@lumc.nl)

**Table S1. Number and frequencies of missing in each variable (N = 318,734)**

**Table S2. The baseline characteristics in Estonian Biobank and Copenhagen General Population Study**

**Table S3. Genome-wide significant SNPs for the SNP-age interaction term (P < 5e-8)**

**Table S4. The interactions between five lead genetic variants and age in women and men from the UK Biobank**

**Table S5. The interactions between five lead genetic variants and age in Estonian Biobank and Copenhagen General Population Study**

**Figure S1. Associations between the genotypes of the lead SNPs and the corresponding phenotypes for age-stratified analyses in women and men from UK Biobank**

**Table S1. Number and frequencies of missing in each variable (N = 318,734)**

| **Variables** | **Number (Percentage)** |
| --- | --- |
| apoprotein B | 16,393 (5.14%) |
| low-density lipoprotein-cholesterol | 15,494 (4.86%) |
| triglycerides | 15,154 (4.75%) |
| systolic blood pressure | 27,794 (8.72%) |
| body mass index | 5,581 (1.75%) |

**Table S2. The baseline characteristics in Estonian Biobank and Copenhagen General Population Study.**

| **Estonian Biobank** | | | | | | | |  |
| --- | --- | --- | --- | --- | --- | --- | --- | --- |
|  | **N (n (%) men)** | **Overall** | **20-39** | **40-49** | **50-59** | **60-69** | **70-80** |  |
| SBP (mmHg), mean (SD) | 83849 (34.7%) | 126 (17.1) | 119 (13.8) | 125 (15.8) | 132 (17) | 137 (17.1) | 140 (17.5) |  |
| BMI (kg/m^2^), mean (SD) | 99219 (34.8%) | 26.5 (5.50) | 24.6 (4.93) | 26.9 (5.49) | 28.1 (5.53) | 28.6 (5.49) | 27.9 (5.11) |  |
| LDL-C (mmol/L),  mean (SD) | 97279 (34.4%) | 2.10 (0.576) | 1.88 (0.511) | 2.15 (0.545) | 2.32 (0.578) | 2.28 (0.576) | 2.18 (0.592) |  |
| TG (mmol/L),  median [IQR] | 97283 (34.4%) | 1.34 (1.01) | 1.12 (0.79) | 1.34 (1.03) | 1.53 (1.11) | 1.61 (1.07) | 1.54 (0.97) |  |
| ApoB (g/L), mean (SD) | 97283 (34.4%) | 0.97 (0.26) | 0.86 (0.23) | 0.99 (0.24) | 1.07 (0.26) | 1.07 (0.26) | 1.04 (0.26) |  |
| **Copenhagen General Population Study** | | | | | | | |  |
|  | **N (n (%) men)** | **Overall** | **20-39** | **40-49** | **50-59** | **60-69** | **70-79** | **80-110** |
| SBP (mmHg), mean (SD) | 108350 (44.9%) | 141.49 (21.38) | 128.41 (15.92) | 132.64 (17.88) | 139.86 (20.17) | 146.61 (21.07) | 151.18 (21.53) | 154.44 (22.47) |
| BMI (kg/m^2^), mean (SD) | 108217 (44.9%) | 26.14 (4.27) | 24.86 (4.24) | 25.65 (4.21) | 26.24 (4.32) | 26.61 (4.30) | 26.59 (4.14) | 25.87 (3.78) |
| LDL-C (mmol/L),  mean (SD) | 106885(44.9%) | 3.35 (0.95) | 2.81 (0.85) | 3.17 (0.90) | 3.48 (0.94) | 3.53 (0.95) | 3.40 (0.93) | 3.29 (0.95) |
| TG (mmol/L),  median [IQR] | 107504 (44.9%) | 1.38  [0.96, 2.05] | 1.12  [0.81, 1.69] | 1.24  [0.86, 1.90] | 1.40  [0.97, 2.11] | 1.48  [1.04, 2.15] | 1.48  [1.05, 2.11] | 1.36  [0.99, 1.95] |
| ApoB (g/L), mean (SD) | 107435 (44.9%) | 1.10 (0.33) | 0.94 (0.31) | 1.05 (0.34) | 1.15 (0.34) | 1.14 (0.32) | 1.09 (0.31) | 1.05 (0.30) |

Abbreviations: ApoB, apolipoprotein B; BMI, body mass index; IQR, interquartile range; LDL-C, low-density lipoprotein cholesterol; SBP, systolic blood pressure; SD, standard deviation; TG, triglyceride.

**Table S3. Genome-wide significant SNPs for the SNP-age interaction term (*P* < 5e-8).**

| **RSID** | **CHR** | **POS** | **Non Effect Allele** | **Effect Allele** | **AF** | **Beta G-Age** | **Robust SE Beta-G-Age** | **Robust P Value Interaction** | **Phenotype** |
| --- | --- | --- | --- | --- | --- | --- | --- | --- | --- |
| rs11591147 | 1 | 55505647 | G | T | 0.017401 | 0.001846 | 0.000314 | 3.95E-09 | ApoB |
| rs143020224 | 19 | 11187324 | C | G | 0.118749 | 0.000693 | 0.000122 | 1.43E-08 | ApoB |
| rs144826254 | 19 | 11187358 | T | G | 0.118633 | 0.000694 | 0.000122 | 1.37E-08 | ApoB |
| rs112736558 | 19 | 11187422 | T | C | 0.118765 | 0.000695 | 0.000122 | 1.30E-08 | ApoB |
| rs111989435 | 19 | 11187611 | A | G | 0.118751 | 0.000693 | 0.000122 | 1.40E-08 | ApoB |
| rs55997232 | 19 | 11188117 | C | T | 0.118505 | 0.000693 | 0.000122 | 1.41E-08 | ApoB |
| rs55791371 | 19 | 11188153 | A | C | 0.118516 | 0.000693 | 0.000122 | 1.39E-08 | ApoB |
| rs56125973 | 19 | 11188164 | T | C | 0.1185 | 0.000696 | 0.000122 | 1.20E-08 | ApoB |
| rs56289821 | 19 | 11188247 | G | A | 0.118446 | 0.000691 | 0.000122 | 1.54E-08 | ApoB |
| rs145329186 | 19 | 11188313 | C | CTTTA | 0.118893 | 0.000679 | 0.000122 | 2.68E-08 | ApoB |
| rs112898275 | 19 | 11188850 | T | C | 0.118655 | 0.000693 | 0.000122 | 1.34E-08 | ApoB |
| rs112374545 | 19 | 11188899 | C | T | 0.118669 | 0.000696 | 0.000122 | 1.19E-08 | ApoB |
| 19:11188974_AC_A | 19 | 11188974 | AC | A | 0.118856 | 0.000712 | 0.000122 | 5.63E-09 | ApoB |
| rs148898583 | 19 | 11189205 | C | G | 0.118614 | 0.000705 | 0.000122 | 7.73E-09 | ApoB |
| rs113722226 | 19 | 11189272 | T | C | 0.118752 | 0.000702 | 0.000122 | 8.73E-09 | ApoB |
| rs73015011 | 19 | 11189764 | T | C | 0.118785 | 0.000707 | 0.000122 | 6.99E-09 | ApoB |
| rs114821903 | 19 | 11189937 | T | A | 0.118845 | 0.000705 | 0.000122 | 7.69E-09 | ApoB |
| rs138175288 | 19 | 11189980 | C | A | 0.118839 | 0.000705 | 0.000122 | 7.59E-09 | ApoB |
| rs112107114 | 19 | 11190074 | G | A | 0.11886 | 0.000705 | 0.000122 | 7.46E-09 | ApoB |
| rs115594766 | 19 | 11190110 | A | G | 0.118859 | 0.000705 | 0.000122 | 7.41E-09 | ApoB |
| rs112032422 | 19 | 11190292 | T | C | 0.118581 | 0.000703 | 0.000122 | 8.54E-09 | ApoB |
| rs77265569 | 19 | 11190481 | G | T | 0.11349 | 0.000714 | 0.000126 | 1.40E-08 | ApoB |
| rs142158911 | 19 | 11190534 | G | A | 0.116034 | 0.000701 | 0.000124 | 1.64E-08 | ApoB |
| rs142130958 | 19 | 11190652 | G | A | 0.118756 | 0.000707 | 0.000122 | 6.85E-09 | ApoB |
| rs73015013 | 19 | 11190873 | C | T | 0.118865 | 0.000706 | 0.000122 | 7.20E-09 | ApoB |
| rs114846969 | 19 | 11191197 | G | A | 0.117115 | 0.000706 | 0.000123 | 1.04E-08 | ApoB |
| rs151113958 | 19 | 11191201 | A | AG | 0.118181 | 0.000682 | 0.000123 | 2.89E-08 | ApoB |
| rs73015016 | 19 | 11191300 | G | A | 0.119466 | 0.000691 | 0.000122 | 1.34E-08 | ApoB |
| rs10402112 | 19 | 11191677 | T | A | 0.119233 | 0.000696 | 0.000122 | 1.10E-08 | ApoB |
| rs138294113 | 19 | 11191729 | C | T | 0.118754 | 0.000708 | 0.000122 | 6.44E-09 | ApoB |
| rs61194703 | 19 | 11192193 | A | T | 0.119285 | 0.000705 | 0.000122 | 7.06E-09 | ApoB |
| rs73015020 | 19 | 11192550 | G | A | 0.119339 | 0.000703 | 0.000122 | 7.67E-09 | ApoB |
| rs77140532 | 19 | 11192831 | A | G | 0.119719 | 0.000698 | 0.000122 | 9.39E-09 | ApoB |
| rs375484700 | 19 | 11192876 | C | CT | 0.119247 | 0.000698 | 0.000122 | 1.18E-08 | ApoB |
| rs73015021 | 19 | 11192915 | A | G | 0.119714 | 0.000697 | 0.000122 | 9.67E-09 | ApoB |
| rs112552009 | 19 | 11193091 | T | G | 0.11868 | 0.00071 | 0.000122 | 6.09E-09 | ApoB |
| rs10412048 | 19 | 11193949 | A | G | 0.119972 | 0.000694 | 0.000121 | 1.12E-08 | ApoB |
| 19:11196356_AC_A | 19 | 11196356 | AC | A | 0.118863 | 0.000716 | 0.000122 | 4.45E-09 | ApoB |
| 19:11196651_AT_A | 19 | 11196651 | AT | A | 0.144343 | 0.000662 | 0.000117 | 1.76E-08 | ApoB |
| rs8106503 | 19 | 11196886 | T | C | 0.1178 | 0.000731 | 0.000122 | 2.38E-09 | ApoB |
| rs12151108 | 19 | 11197261 | G | A | 0.118874 | 0.000718 | 0.000122 | 3.92E-09 | ApoB |
| rs73015024 | 19 | 11197598 | G | T | 0.118866 | 0.000719 | 0.000122 | 3.76E-09 | ApoB |
| rs147985405 | 19 | 11197750 | G | GA | 0.118578 | 0.000711 | 0.000122 | 5.83E-09 | ApoB |
| rs17248720 | 19 | 11198187 | C | T | 0.117916 | 0.000735 | 0.000122 | 1.97E-09 | ApoB |
| rs17248727 | 19 | 11198502 | T | C | 0.119124 | 0.000717 | 0.000122 | 4.14E-09 | ApoB |
| rs57217136 | 19 | 11201124 | T | C | 0.1195 | 0.000715 | 0.000122 | 4.29E-09 | ApoB |
| 19:11201988_GT_G | 19 | 11201988 | GT | G | 0.119164 | 0.000715 | 0.000122 | 4.41E-09 | ApoB |
| 19:11202194_GC_G | 19 | 11202194 | GC | G | 0.119221 | 0.000722 | 0.000122 | 3.02E-09 | ApoB |
| rs6511720 | 19 | 11202306 | G | T | 0.119021 | 0.000719 | 0.000122 | 3.70E-09 | ApoB |
| rs142042446 | 19 | 45386467 | G | GTAA | 0.147226 | -0.00072 | 0.000117 | 1.05E-09 | ApoB |
| rs12972156 | 19 | 45387459 | C | G | 0.146989 | -0.00074 | 0.000116 | 2.28E-10 | ApoB |
| rs12972970 | 19 | 45387596 | G | A | 0.147101 | -0.00074 | 0.000116 | 2.04E-10 | ApoB |
| rs34342646 | 19 | 45388130 | G | A | 0.150454 | -0.00073 | 0.000115 | 3.04E-10 | ApoB |
| rs6857 | 19 | 45392254 | C | T | 0.169786 | -0.00069 | 0.000109 | 3.38E-10 | ApoB |
| rs71352238 | 19 | 45394336 | T | C | 0.147542 | -0.00073 | 0.000116 | 3.51E-10 | ApoB |
| rs2075650 | 19 | 45395619 | A | G | 0.144624 | -0.00073 | 0.000117 | 3.50E-10 | ApoB |
| rs34404554 | 19 | 45395909 | C | G | 0.144545 | -0.00073 | 0.000117 | 4.81E-10 | ApoB |
| rs11556505 | 19 | 45396144 | C | T | 0.144561 | -0.00073 | 0.000117 | 3.88E-10 | ApoB |
| rs769449 | 19 | 45410002 | G | A | 0.124626 | -0.0009 | 0.000125 | 6.03E-13 | ApoB |
| rs429358 | 19 | 45411941 | T | C | 0.153809 | -0.00085 | 0.000114 | 9.00E-14 | ApoB |
| rs7412 | 19 | 45412079 | C | T | 0.079676 | 0.001005 | 0.000176 | 1.06E-08 | ApoB |
| rs1065853 | 19 | 45413233 | G | T | 0.079513 | 0.001009 | 0.000176 | 1.04E-08 | ApoB |
| 19:45413234_GGT_G | 19 | 45413234 | GGT | G | 0.134415 | -0.00092 | 0.000129 | 1.03E-12 | ApoB |
| rs10414043 | 19 | 45415713 | G | A | 0.126246 | -0.00088 | 0.000125 | 1.29E-12 | ApoB |
| rs7256200 | 19 | 45415935 | G | T | 0.126358 | -0.00088 | 0.000125 | 1.28E-12 | ApoB |
| rs12721046 | 19 | 45421254 | G | A | 0.156705 | -0.00066 | 0.000113 | 4.15E-09 | ApoB |
| rs12721051 | 19 | 45422160 | C | G | 0.188413 | -0.00058 | 0.000105 | 3.60E-08 | ApoB |
| rs56131196 | 19 | 45422846 | G | A | 0.188642 | -0.00058 | 0.000105 | 2.96E-08 | ApoB |
| rs190712692 | 19 | 45425178 | G | A | 0.052977 | 0.001169 | 0.000213 | 4.23E-08 | ApoB |
| rs111789331 | 19 | 45427125 | T | A | 0.15797 | -0.00069 | 0.000114 | 1.93E-09 | ApoB |
| rs66626994 | 19 | 45428234 | G | A | 0.157624 | -0.00069 | 0.000115 | 1.78E-09 | ApoB |
| rs62122481 | 2 | 21216815 | C | A | 0.377134 | -0.00046 | 8.37E-05 | 3.44E-08 | ApoB |
| rs312944 | 2 | 21325188 | A | T | 0.803709 | -0.00055 | 0.0001 | 3.94E-08 | ApoB |
| rs312953 | 2 | 21339932 | G | A | 0.803856 | -0.00055 | 0.0001 | 4.74E-08 | ApoB |
| 2:21371195_CTCT_C | 2 | 21371195 | CTCT | C | 0.803943 | -0.00055 | 0.0001 | 3.71E-08 | ApoB |
| rs34601365 | 2 | 21371930 | C | CT | 0.791856 | -0.00058 | 0.000101 | 8.38E-09 | ApoB |
| 2:21372270_CTT_C | 2 | 21372270 | CTT | C | 0.814056 | -0.00057 | 0.000104 | 3.49E-08 | ApoB |
| rs312979 | 2 | 21377705 | A | T | 0.803793 | -0.00055 | 0.0001 | 4.24E-08 | ApoB |
| rs35425016 | 2 | 21378013 | C | CA | 0.804663 | -0.00056 | 0.0001 | 2.61E-08 | ApoB |
| rs312981 | 2 | 21378151 | G | A | 0.803796 | -0.00055 | 0.0001 | 4.19E-08 | ApoB |
| rs312982 | 2 | 21378226 | G | C | 0.803781 | -0.00055 | 0.0001 | 4.06E-08 | ApoB |
| 2:21378431_CT_C | 2 | 21378431 | CT | C | 0.8038 | -0.00055 | 0.0001 | 4.27E-08 | ApoB |
| 2:21378559_GT_G | 2 | 21378559 | GT | G | 0.805736 | -0.00055 | 0.000101 | 4.25E-08 | ApoB |
| rs312983 | 2 | 21378580 | A | C | 0.803798 | -0.00055 | 0.0001 | 4.17E-08 | ApoB |
| rs312984 | 2 | 21378778 | C | T | 0.8038 | -0.00055 | 0.0001 | 4.07E-08 | ApoB |
| rs312985 | 2 | 21378805 | A | G | 0.803803 | -0.00055 | 0.0001 | 4.10E-08 | ApoB |
| rs756699008 | 2 | 21379144 | CAACTA | C | 0.80391 | -0.00055 | 0.0001 | 3.75E-08 | ApoB |
| rs529396 | 2 | 21381268 | G | C | 0.803789 | -0.00055 | 0.0001 | 4.09E-08 | ApoB |
| rs5829773 | 2 | 21381292 | A | AAAAC | 0.803546 | -0.00055 | 0.0001 | 3.76E-08 | ApoB |
| rs530474 | 2 | 21381435 | G | A | 0.803791 | -0.00055 | 0.0001 | 4.08E-08 | ApoB |
| rs559318 | 2 | 21381490 | C | T | 0.8038 | -0.00055 | 0.0001 | 4.21E-08 | ApoB |
| rs532300 | 2 | 21381608 | C | A | 0.8038 | -0.00055 | 0.0001 | 4.21E-08 | ApoB |
| rs558130 | 2 | 21381689 | T | G | 0.8038 | -0.00055 | 0.0001 | 4.21E-08 | ApoB |
| rs533211 | 2 | 21381702 | G | A | 0.8038 | -0.00055 | 0.0001 | 4.21E-08 | ApoB |
| rs557197 | 2 | 21381781 | T | G | 0.8038 | -0.00055 | 0.0001 | 4.21E-08 | ApoB |
| rs34416543 | 2 | 21382105 | G | GA | 0.803917 | -0.00055 | 0.0001 | 4.28E-08 | ApoB |
| rs11388395 | 2 | 21382319 | C | CT | 0.803672 | -0.00055 | 0.0001 | 4.37E-08 | ApoB |
| rs560522 | 2 | 21382363 | A | C | 0.803806 | -0.00055 | 0.0001 | 4.24E-08 | ApoB |
| rs528114 | 2 | 21382623 | T | G | 0.803802 | -0.00055 | 0.0001 | 4.18E-08 | ApoB |
| rs528113 | 2 | 21382624 | G | T | 0.803806 | -0.00055 | 0.0001 | 4.23E-08 | ApoB |
| rs527034 | 2 | 21382786 | C | A | 0.803805 | -0.00055 | 0.0001 | 4.18E-08 | ApoB |
| rs525172 | 2 | 21382976 | T | G | 0.803756 | -0.00055 | 0.0001 | 4.11E-08 | ApoB |
| rs479413 | 2 | 21383279 | T | G | 0.803809 | -0.00055 | 0.0001 | 4.20E-08 | ApoB |
| rs480488 | 2 | 21383434 | A | G | 0.803803 | -0.00055 | 0.0001 | 4.17E-08 | ApoB |
| rs1712246 | 2 | 21383514 | G | A | 0.804383 | -0.00056 | 0.0001 | 2.85E-08 | ApoB |
| rs1652423 | 2 | 21383524 | G | A | 0.804383 | -0.00056 | 0.0001 | 2.86E-08 | ApoB |
| rs4560142 | 2 | 21383717 | C | T | 0.803838 | -0.00055 | 0.0001 | 3.71E-08 | ApoB |
| rs4591370 | 2 | 21383742 | A | G | 0.803836 | -0.00055 | 0.0001 | 3.70E-08 | ApoB |
| rs1652422 | 2 | 21383841 | A | G | 0.80383 | -0.00055 | 0.0001 | 3.68E-08 | ApoB |
| rs1652421 | 2 | 21383848 | A | G | 0.80383 | -0.00055 | 0.0001 | 3.68E-08 | ApoB |
| rs1652420 | 2 | 21383881 | A | T | 0.803862 | -0.00055 | 0.0001 | 3.56E-08 | ApoB |
| rs1712247 | 2 | 21383951 | C | T | 0.803837 | -0.00055 | 0.0001 | 3.71E-08 | ApoB |
| rs540897 | 2 | 21383982 | A | G | 0.802346 | -0.00055 | 9.99E-05 | 4.04E-08 | ApoB |
| rs492255 | 2 | 21384272 | C | T | 0.803832 | -0.00055 | 0.0001 | 3.67E-08 | ApoB |
| rs544450 | 2 | 21384358 | T | C | 0.803803 | -0.00055 | 0.0001 | 3.74E-08 | ApoB |
| rs547179 | 2 | 21384662 | A | G | 0.804205 | -0.00055 | 0.0001 | 4.17E-08 | ApoB |
| rs547235 | 2 | 21384682 | A | G | 0.803958 | -0.00055 | 0.0001 | 3.75E-08 | ApoB |
| rs547239 | 2 | 21384686 | T | A | 0.804362 | -0.00055 | 0.0001 | 3.99E-08 | ApoB |
| rs572246 | 2 | 21385149 | T | C | 0.803838 | -0.00055 | 0.0001 | 3.72E-08 | ApoB |
| rs573314 | 2 | 21385161 | C | G | 0.803836 | -0.00055 | 0.0001 | 3.74E-08 | ApoB |
| rs548506 | 2 | 21385541 | A | G | 0.803712 | -0.00055 | 0.0001 | 3.65E-08 | ApoB |
| rs1652419 | 2 | 21385649 | C | T | 0.803782 | -0.00055 | 0.0001 | 3.42E-08 | ApoB |
| rs1712248 | 2 | 21385778 | C | G | 0.799331 | -0.00055 | 9.93E-05 | 2.89E-08 | ApoB |
| rs1712250 | 2 | 21386058 | C | T | 0.803559 | -0.00055 | 0.0001 | 4.04E-08 | ApoB |
| rs1367120 | 2 | 21386304 | T | C | 0.80376 | -0.00055 | 0.0001 | 3.36E-08 | ApoB |
| rs1367119 | 2 | 21386375 | A | G | 0.803756 | -0.00055 | 0.0001 | 3.60E-08 | ApoB |
| rs522963 | 2 | 21386957 | T | C | 0.803787 | -0.00055 | 0.0001 | 3.31E-08 | ApoB |
| rs522250 | 2 | 21387113 | T | C | 0.803787 | -0.00055 | 0.0001 | 3.31E-08 | ApoB |
| rs529697 | 2 | 21387948 | G | T | 0.80379 | -0.00055 | 0.0001 | 3.25E-08 | ApoB |
| rs490757 | 2 | 21388224 | C | T | 0.80379 | -0.00055 | 0.0001 | 3.25E-08 | ApoB |
| rs11383998 | 2 | 21388401 | G | GA | 0.804428 | -0.00055 | 0.0001 | 3.40E-08 | ApoB |
| rs1652418 | 2 | 21388456 | T | C | 0.80379 | -0.00055 | 0.0001 | 3.25E-08 | ApoB |
| rs538928 | 2 | 21389019 | A | G | 0.803836 | -0.00055 | 0.0001 | 3.36E-08 | ApoB |
| rs560844 | 2 | 21389108 | A | G | 0.803768 | -0.00055 | 0.0001 | 3.03E-08 | ApoB |
| rs563696 | 2 | 21389430 | T | A | 0.803782 | -0.00055 | 0.0001 | 3.26E-08 | ApoB |
| rs475887 | 2 | 21389485 | T | G | 0.803767 | -0.00055 | 0.0001 | 3.03E-08 | ApoB |
| rs479545 | 2 | 21389897 | T | C | 0.803755 | -0.00055 | 0.0001 | 3.02E-08 | ApoB |
| rs501863 | 2 | 21390000 | G | A | 0.803767 | -0.00055 | 0.0001 | 3.15E-08 | ApoB |
| rs480732 | 2 | 21390149 | A | T | 0.803736 | -0.00055 | 0.0001 | 3.38E-08 | ApoB |
| rs480787 | 2 | 21390169 | A | G | 0.803736 | -0.00055 | 0.0001 | 3.37E-08 | ApoB |
| 2:21390309_TA_T | 2 | 21390309 | TA | T | 0.804535 | -0.00056 | 0.0001 | 1.93E-08 | ApoB |
| rs483436 | 2 | 21390407 | G | A | 0.803968 | -0.00055 | 0.0001 | 3.11E-08 | ApoB |
| rs486139 | 2 | 21390674 | G | A | 0.803713 | -0.00055 | 0.0001 | 3.34E-08 | ApoB |
| rs489010 | 2 | 21390992 | G | A | 0.803724 | -0.00055 | 0.0001 | 3.43E-08 | ApoB |
| rs514757 | 2 | 21391531 | G | A | 0.803714 | -0.00055 | 0.0001 | 3.35E-08 | ApoB |
| rs538528 | 2 | 21391740 | T | C | 0.803714 | -0.00055 | 0.0001 | 3.35E-08 | ApoB |
| rs518280 | 2 | 21391892 | G | A | 0.803714 | -0.00055 | 0.0001 | 3.35E-08 | ApoB |
| rs540439 | 2 | 21391978 | C | T | 0.803714 | -0.00055 | 0.0001 | 3.35E-08 | ApoB |
| rs563280 | 2 | 21392153 | T | G | 0.80372 | -0.00055 | 0.0001 | 3.38E-08 | ApoB |
| rs564073 | 2 | 21392213 | A | T | 0.803715 | -0.00055 | 0.0001 | 3.31E-08 | ApoB |
| rs542261 | 2 | 21392229 | G | A | 0.803715 | -0.00055 | 0.0001 | 3.31E-08 | ApoB |
| rs4558611 | 2 | 21392289 | C | G | 0.803782 | -0.00055 | 0.0001 | 3.54E-08 | ApoB |
| rs565894 | 2 | 21392426 | T | C | 0.803721 | -0.00055 | 0.0001 | 3.38E-08 | ApoB |
| rs566913 | 2 | 21392570 | C | T | 0.803717 | -0.00055 | 0.0001 | 3.31E-08 | ApoB |
| rs568740 | 2 | 21392744 | C | T | 0.803714 | -0.00055 | 0.0001 | 3.35E-08 | ApoB |
| rs548594 | 2 | 21392918 | T | C | 0.803714 | -0.00055 | 0.0001 | 3.35E-08 | ApoB |
| rs484802 | 2 | 21393321 | C | T | 0.803716 | -0.00055 | 0.0001 | 3.31E-08 | ApoB |
| rs484906 | 2 | 21393348 | G | T | 0.803715 | -0.00055 | 0.0001 | 3.35E-08 | ApoB |
| rs576203 | 2 | 21393623 | A | G | 0.803721 | -0.00055 | 0.0001 | 3.38E-08 | ApoB |
| rs488507 | 2 | 21393689 | G | T | 0.803721 | -0.00055 | 0.0001 | 3.34E-08 | ApoB |
| rs578095 | 2 | 21393866 | A | G | 0.80372 | -0.00055 | 0.0001 | 3.40E-08 | ApoB |
| rs578864 | 2 | 21393937 | C | G | 0.803719 | -0.00055 | 0.0001 | 3.35E-08 | ApoB |
| rs492364 | 2 | 21394199 | C | T | 0.803718 | -0.00055 | 0.0001 | 3.40E-08 | ApoB |
| rs492365 | 2 | 21394201 | A | G | 0.80372 | -0.00055 | 0.0001 | 3.36E-08 | ApoB |
| rs492494 | 2 | 21394248 | C | T | 0.803719 | -0.00055 | 0.0001 | 3.39E-08 | ApoB |
| rs493404 | 2 | 21394340 | G | A | 0.803721 | -0.00055 | 0.0001 | 3.45E-08 | ApoB |
| rs34345287 | 2 | 21394368 | A | AAAG | 0.804698 | -0.00055 | 0.0001 | 3.51E-08 | ApoB |
| rs549959 | 2 | 21394458 | T | C | 0.803719 | -0.00055 | 0.0001 | 3.39E-08 | ApoB |
| rs496100 | 2 | 21394614 | A | G | 0.803532 | -0.00055 | 0.0001 | 3.75E-08 | ApoB |
| rs553523 | 2 | 21394798 | T | G | 0.803703 | -0.00055 | 0.0001 | 3.23E-08 | ApoB |
| rs554414 | 2 | 21394892 | T | C | 0.803721 | -0.00055 | 0.0001 | 3.35E-08 | ApoB |
| rs556504 | 2 | 21395261 | C | G | 0.80372 | -0.00055 | 0.0001 | 3.39E-08 | ApoB |
| rs557316 | 2 | 21395337 | A | G | 0.803721 | -0.00055 | 0.0001 | 3.35E-08 | ApoB |
| rs558342 | 2 | 21395469 | G | A | 0.80372 | -0.00055 | 0.0001 | 3.39E-08 | ApoB |
| rs561850 | 2 | 21395805 | A | G | 0.80372 | -0.00055 | 0.0001 | 3.39E-08 | ApoB |
| rs473269 | 2 | 21395830 | T | C | 0.80372 | -0.00055 | 0.0001 | 3.39E-08 | ApoB |
| rs563719 | 2 | 21396001 | C | A | 0.803721 | -0.00055 | 0.0001 | 3.35E-08 | ApoB |
| rs563752 | 2 | 21396013 | A | G | 0.80372 | -0.00055 | 0.0001 | 3.39E-08 | ApoB |
| rs477146 | 2 | 21396334 | A | G | 0.80372 | -0.00055 | 0.0001 | 3.39E-08 | ApoB |
| rs13411597 | 2 | 21396488 | G | A | 0.803721 | -0.00055 | 0.0001 | 3.35E-08 | ApoB |
| rs1652417 | 2 | 21396517 | T | C | 0.803715 | -0.00055 | 0.0001 | 3.42E-08 | ApoB |
| rs1712251 | 2 | 21396551 | G | A | 0.803721 | -0.00055 | 0.0001 | 3.35E-08 | ApoB |
| rs1652416 | 2 | 21396609 | A | G | 0.803721 | -0.00055 | 0.0001 | 3.35E-08 | ApoB |
| rs483621 | 2 | 21397035 | T | A | 0.803718 | -0.00055 | 0.0001 | 3.38E-08 | ApoB |
| rs506585 | 2 | 21397182 | G | A | 0.803719 | -0.00055 | 0.0001 | 3.34E-08 | ApoB |
| rs507616 | 2 | 21397321 | G | C | 0.803717 | -0.00055 | 0.0001 | 3.38E-08 | ApoB |
| rs531380 | 2 | 21397561 | C | G | 0.803719 | -0.00055 | 0.0001 | 3.34E-08 | ApoB |
| rs532225 | 2 | 21397637 | T | C | 0.803681 | -0.00055 | 0.0001 | 3.42E-08 | ApoB |
| rs560408 | 2 | 21398379 | T | C | 0.803849 | -0.00055 | 0.0001 | 2.99E-08 | ApoB |
| rs541569 | 2 | 21398768 | G | A | 0.803848 | -0.00055 | 0.0001 | 3.04E-08 | ApoB |
| 2:21398857_GA_G | 2 | 21398857 | GA | G | 0.803959 | -0.00055 | 0.0001 | 3.05E-08 | ApoB |
| rs544039 | 2 | 21398985 | C | A | 0.803848 | -0.00055 | 0.0001 | 3.04E-08 | ApoB |
| rs478442 | 2 | 21399216 | G | T | 0.803848 | -0.00055 | 0.0001 | 3.04E-08 | ApoB |
| rs570033 | 2 | 21399567 | C | G | 0.803848 | -0.00055 | 0.0001 | 3.04E-08 | ApoB |
| rs574438 | 2 | 21399961 | T | C | 0.803818 | -0.00055 | 0.0001 | 3.37E-08 | ApoB |
| 2:21399964_AT_A | 2 | 21399964 | AT | A | 0.798694 | -0.00058 | 0.0001 | 8.93E-09 | ApoB |
| rs574461 | 2 | 21400013 | A | G | 0.80385 | -0.00055 | 0.0001 | 3.04E-08 | ApoB |
| rs487858 | 2 | 21400305 | G | A | 0.803855 | -0.00055 | 0.0001 | 3.06E-08 | ApoB |
| rs494315 | 2 | 21400950 | C | T | 0.803852 | -0.00055 | 0.0001 | 3.04E-08 | ApoB |
| rs494465 | 2 | 21401010 | C | T | 0.803855 | -0.00055 | 0.0001 | 3.01E-08 | ApoB |
| rs522822 | 2 | 21401851 | A | G | 0.803832 | -0.00055 | 0.0001 | 2.98E-08 | ApoB |
| rs502323 | 2 | 21403297 | A | C | 0.803851 | -0.00055 | 0.0001 | 3.01E-08 | ApoB |
| rs504091 | 2 | 21403481 | T | G | 0.803848 | -0.00055 | 0.0001 | 2.95E-08 | ApoB |
| rs486246 | 2 | 21406751 | G | A | 0.803771 | -0.00056 | 0.0001 | 2.86E-08 | ApoB |
| rs312936 | 2 | 21408647 | T | A | 0.803842 | -0.00055 | 0.0001 | 3.12E-08 | ApoB |
| rs142786027 | 2 | 21413178 | G | C | 0.8038 | -0.00055 | 0.0001 | 3.83E-08 | ApoB |
| rs141479646 | 2 | 21413218 | A | G | 0.803832 | -0.00055 | 0.0001 | 3.86E-08 | ApoB |
| rs138905573 | 2 | 21413284 | A | C | 0.803713 | -0.00055 | 0.0001 | 3.95E-08 | ApoB |
| rs1712252 | 2 | 21413738 | G | A | 0.80362 | -0.00055 | 0.0001 | 3.54E-08 | ApoB |
| rs504616 | 2 | 21414020 | C | G | 0.803802 | -0.00055 | 0.0001 | 4.13E-08 | ApoB |
| rs2435386 | 2 | 21414760 | C | T | 0.800341 | -0.00055 | 9.99E-05 | 4.57E-08 | ApoB |
| rs569014 | 2 | 21415763 | G | A | 0.803375 | -0.00056 | 0.0001 | 2.70E-08 | ApoB |
| rs527259 | 2 | 21416019 | G | C | 0.803321 | -0.00055 | 0.0001 | 3.15E-08 | ApoB |
| rs141187066 | 2 | 21416345 | G | GAAC | 0.802813 | -0.00055 | 0.0001 | 3.61E-08 | ApoB |
| rs62133415 | 2 | 21416346 | T | A | 0.80314 | -0.00055 | 0.0001 | 3.05E-08 | ApoB |
| rs769304839 | 2 | 21416347 | GT | G | 0.802813 | -0.00055 | 0.0001 | 3.61E-08 | ApoB |
| rs539845 | 2 | 21416614 | A | T | 0.804807 | -0.00058 | 0.0001 | 1.03E-08 | ApoB |
| rs4596008 | 2 | 21417576 | C | T | 0.1952 | 0.000552 | 0.0001 | 3.83E-08 | ApoB |
| rs67599264 | 2 | 21418083 | G | A | 0.194399 | 0.000549 | 0.000101 | 4.65E-08 | ApoB |
| rs36047821 | 2 | 21418719 | C | A | 0.195092 | 0.000553 | 0.0001 | 3.49E-08 | ApoB |
| rs34125138 | 2 | 21418756 | A | G | 0.195087 | 0.000554 | 0.0001 | 3.42E-08 | ApoB |
| rs11897480 | 2 | 21419320 | C | G | 0.195124 | 0.000554 | 0.0001 | 3.35E-08 | ApoB |
| rs10221742 | 2 | 21419650 | G | A | 0.240585 | 0.000523 | 9.45E-05 | 3.16E-08 | ApoB |
| rs10221876 | 2 | 21419809 | A | T | 0.195209 | 0.000554 | 0.0001 | 3.42E-08 | ApoB |
| rs1878512 | 2 | 21420358 | C | A | 0.195211 | 0.000554 | 0.0001 | 3.35E-08 | ApoB |
| rs17041988 | 2 | 21420697 | A | G | 0.195262 | 0.000554 | 0.0001 | 3.45E-08 | ApoB |
| rs12712923 | 2 | 21421782 | C | A | 0.195156 | 0.000555 | 0.0001 | 3.28E-08 | ApoB |
| rs7578527 | 2 | 21422403 | G | A | 0.195166 | 0.000557 | 0.0001 | 2.98E-08 | ApoB |
| rs17042000 | 2 | 21422928 | C | T | 0.194275 | 0.000553 | 0.000101 | 4.07E-08 | ApoB |
| rs10169543 | 2 | 21423989 | T | C | 0.195763 | 0.000564 | 0.0001 | 1.92E-08 | ApoB |
| rs34002646 | 2 | 21425323 | A | G | 0.195903 | 0.000563 | 0.0001 | 2.05E-08 | ApoB |
| 2:21426683_AAAAG_A | 2 | 21426683 | AAAAG | A | 0.192874 | 0.000554 | 0.000102 | 4.85E-08 | ApoB |
| rs12712940 | 2 | 21426743 | C | A | 0.195711 | 0.000564 | 0.0001 | 2.01E-08 | ApoB |
| rs28601761 | 8 | 1.27E+08 | C | G | 0.419322 | 0.000457 | 8.21E-05 | 2.64E-08 | ApoB |
| rs142042446 | 19 | 45386467 | G | GTAA | 0.147226 | -0.00165 | 0.000245 | 1.74E-11 | TG |
| rs12972156 | 19 | 45387459 | C | G | 0.146989 | -0.00165 | 0.000243 | 1.09E-11 | TG |
| rs12972970 | 19 | 45387596 | G | A | 0.147101 | -0.00164 | 0.000243 | 1.31E-11 | TG |
| rs34342646 | 19 | 45388130 | G | A | 0.150454 | -0.0015 | 0.000241 | 4.84E-10 | TG |
| rs6857 | 19 | 45392254 | C | T | 0.169786 | -0.00155 | 0.000228 | 1.12E-11 | TG |
| rs71352238 | 19 | 45394336 | T | C | 0.147542 | -0.00146 | 0.000242 | 1.44E-09 | TG |
| rs2075650 | 19 | 45395619 | A | G | 0.144624 | -0.00155 | 0.000243 | 1.78E-10 | TG |
| rs34404554 | 19 | 45395909 | C | G | 0.144545 | -0.00156 | 0.000243 | 1.39E-10 | TG |
| rs11556505 | 19 | 45396144 | C | T | 0.144561 | -0.00156 | 0.000243 | 1.55E-10 | TG |
| rs59007384 | 19 | 45396665 | G | T | 0.205556 | -0.00116 | 0.000213 | 4.81E-08 | TG |
| rs769449 | 19 | 45410002 | G | A | 0.124626 | -0.00204 | 0.00026 | 4.03E-15 | TG |
| rs429358 | 19 | 45411941 | T | C | 0.153809 | -0.00193 | 0.000238 | 5.35E-16 | TG |
| 19:45413234_GGT_G | 19 | 45413234 | GGT | G | 0.134415 | -0.00209 | 0.000268 | 5.49E-15 | TG |
| rs10414043 | 19 | 45415713 | G | A | 0.126246 | -0.002 | 0.000259 | 1.05E-14 | TG |
| rs7256200 | 19 | 45415935 | G | T | 0.126358 | -0.00202 | 0.000259 | 6.89E-15 | TG |
| rs12721046 | 19 | 45421254 | G | A | 0.156705 | -0.00153 | 0.000235 | 8.48E-11 | TG |
| rs12721051 | 19 | 45422160 | C | G | 0.188413 | -0.00152 | 0.000219 | 4.26E-12 | TG |
| rs56131196 | 19 | 45422846 | G | A | 0.188642 | -0.00152 | 0.000219 | 3.64E-12 | TG |
| rs4420638 | 19 | 45422946 | A | G | 0.188887 | -0.00152 | 0.000218 | 3.49E-12 | TG |
| rs814573 | 19 | 45424351 | A | T | 0.185682 | -0.00156 | 0.000226 | 5.10E-12 | TG |
| rs157592 | 19 | 45424514 | A | C | 0.184524 | -0.00157 | 0.000226 | 3.93E-12 | TG |
| rs111789331 | 19 | 45427125 | T | A | 0.15797 | -0.00157 | 0.000239 | 5.95E-11 | TG |
| rs66626994 | 19 | 45428234 | G | A | 0.157624 | -0.00159 | 0.000241 | 4.39E-11 | TG |
| rs71313136 | 22 | 33038283 | T | A | 0.028389 | -0.02723 | 0.004913 | 2.99E-08 | BMI |

**Table S4. The interaction effects between five lead genetic variants and age in women and men from the UK Biobank**

| **Estimate** | **Std. Error** | **t value** | ***P* values** | **SNPs** | **Population** | **Trait** |
| --- | --- | --- | --- | --- | --- | --- |
| 0.001313 | 0.000415 | 3.161673 | 0.001569 | rs11591147 | Women | ApoB |
| 0.002497 | 0.000445 | 5.606261 | 2.07e-08 | rs11591147 | Men | ApoB |
| -0.00049 | 0.000137 | -3.57066 | 0.000356 | rs34601365 | Women | ApoB |
| -0.0007 | 0.000148 | -4.70442 | 2.55e-06 | rs34601365 | Men | ApoB |
| 0.000179 | 0.000168 | 1.066 | 0.286425 | rs17248720 | Women | ApoB |
| 0.001351 | 0.000181 | 7.45773 | 8.86e-14 | rs17248720 | Men | ApoB |
| -0.00042 | 0.000149 | -2.83833 | 0.004536 | rs429358 | Women | ApoB |
| -0.00125 | 0.000161 | -7.75622 | 8.82e-15 | rs429358 | Men | ApoB |
| -0.00146 | 0.000303 | -4.82899 | 1.37e-06 | rs429358 | Women | TG |
| -0.00241 | 0.000354 | -6.8093 | 9.85e-12 | rs429358 | Men | TG |

Abbreviations: ApoB, apolipoprotein B; SNP, single-nucleotide polymorphisms; TG, triglyceride.

**Table S5. The interaction effects between the lead genetic variants and age in Estonian Biobank and Copenhagen General Population Study**

| **Estimate** | **Std. Error** | **t value** | ***P* values** | **SNPs** | **Population** | **Trait** | **Study** | **Age** |
| --- | --- | --- | --- | --- | --- | --- | --- | --- |
| 0.000166 | 0.000291 | 0.569803 | 0.568813 | rs11591147 | All | ApoB | EstBB | 20 ~ 80 |
| 3.16e-05 | 0.00035 | 0.090127 | 0.928186 | rs11591147 | Women | ApoB | EstBB | 20 ~ 80 |
| 0.000384 | 0.000513 | 0.7491 | 0.453802 | rs11591147 | Men | ApoB | EstBB | 20 ~ 80 |
| -7.35e-05 | 0.000132 | -0.55831 | 0.576634 | rs17248720 | All | ApoB | EstBB | 20 ~ 80 |
| -0.00022 | 0.000159 | -1.38316 | 0.16662 | rs17248720 | Women | ApoB | EstBB | 20 ~ 80 |
| 0.000268 | 0.00023 | 1.166721 | 0.243331 | rs17248720 | Men | ApoB | EstBB | 20 ~ 80 |
| 1.66e-05 | 7.63e-05 | 0.217409 | 0.82789 | rs62122481 | All | ApoB | EstBB | 20 ~ 80 |
| 0.000164 | 9.18E-05 | 1.787519 | 0.073858 | rs62122481 | Women | ApoB | EstBB | 20 ~ 80 |
| -0.00021 | 0.000134 | -1.56074 | 0.118594 | rs62122481 | Men | ApoB | EstBB | 20 ~ 80 |
| -0.00044 | 0.000108 | -4.0752 | 4.60e-05 | rs429358 | All | ApoB | EstBB | 20 ~ 80 |
| -0.00018 | 0.00013 | -1.42047 | 0.155476 | rs429358 | Women | ApoB | EstBB | 20 ~ 80 |
| -0.00087 | 0.00019 | -4.5891 | 4.47e-06 | rs429358 | Men | ApoB | EstBB | 20 ~ 80 |
| -0.0009 | 0.000214 | -4.20706 | 2.59e-05 | rs429358 | All | TG | EstBB | 20 ~ 80 |
| -0.00071 | 0.000251 | -2.83095 | 0.004642 | rs429358 | Women | TG | EstBB | 20 ~ 80 |
| -0.00116 | 0.000392 | -2.94667 | 0.003214 | rs429358 | Men | TG | EstBB | 20 ~ 80 |
| 0.001619 | 0.000716 | 2.263012 | 0.023639 | rs11591147 | All | ApoB | EstBB | 40 ~ 70 |
| 0.000639 | 0.000854 | 0.74814 | 0.454381 | rs11591147 | Women | ApoB | EstBB | 40 ~ 70 |
| 0.003154 | 0.001262 | 2.499583 | 0.012442 | rs11591147 | Men | ApoB | EstBB | 40 ~ 70 |
| 0.000642 | 0.00032 | 2.00411 | 0.045063 | rs17248720 | All | ApoB | EstBB | 40 ~ 70 |
| 0.000271 | 0.000385 | 0.70581 | 0.480311 | rs17248720 | Women | ApoB | EstBB | 40 ~ 70 |
| 0.001176 | 0.000557 | 2.111866 | 0.034711 | rs17248720 | Men | ApoB | EstBB | 40 ~ 70 |
| -0.00026 | 0.000187 | -1.38912 | 0.164803 | rs62122481 | All | ApoB | EstBB | 40 ~ 70 |
| 0.000102 | 0.000226 | 0.450189 | 0.652577 | rs62122481 | Women | ApoB | EstBB | 40 ~ 70 |
| -0.00087 | 0.00032 | -2.70955 | 0.006744 | rs62122481 | Men | ApoB | EstBB | 40 ~ 70 |
| -0.00092 | 0.000264 | -3.50429 | 0.000458 | rs429358 | All | ApoB | EstBB | 40 ~ 70 |
| -0.00037 | 0.000319 | -1.16874 | 0.242518 | rs429358 | Women | ApoB | EstBB | 40 ~ 70 |
| -0.0017 | 0.000451 | -3.76352 | 0.000168 | rs429358 | Men | ApoB | EstBB | 40 ~ 70 |
| -0.00114 | 0.000513 | -2.21779 | 0.026573 | rs429358 | All | TG | EstBB | 40 ~ 70 |
| -0.00068 | 0.000602 | -1.12608 | 0.260139 | rs429358 | Women | TG | EstBB | 40 ~ 70 |
| -0.00153 | 0.000933 | -1.64218 | 0.100569 | rs429358 | Men | TG | EstBB | 40 ~ 70 |
| -0.00058 | 0.000149 | -3.91443 | 9.07e-05 | rs429358 | All | ApoB | CGPS | 20 ~ 110 |
| -0.00047 | 0.000188 | -2.50373 | 0.012292 | rs429358 | Women | ApoB | CGPS | 20 ~ 110 |
| -0.00064 | 0.000234 | -2.72499 | 0.006433 | rs429358 | Men | ApoB | CGPS | 20 ~ 110 |
| 0.000268 | 0.000503 | 0.533609 | 0.593613 | rs11591147 | All | ApoB | CGPS | 20 ~ 110 |
| 0.001143 | 0.000639 | 1.788543 | 0.073694 | rs11591147 | Women | ApoB | CGPS | 20 ~ 110 |
| -0.00039 | 0.000779 | -0.50488 | 0.613649 | rs11591147 | Men | ApoB | CGPS | 20 ~ 110 |
| -0.00125 | 0.000242 | -5.16999 | 2.35e-07 | rs429358 | All | TG | CGPS | 20 ~ 110 |
| -0.00117 | 0.000311 | -3.74619 | 0.00018 | rs429358 | Women | TG | CGPS | 20 ~ 110 |
| -0.00128 | 0.000375 | -3.40898 | 0.000653 | rs429358 | Men | TG | CGPS | 20 ~ 110 |
| -0.00098 | 0.000266 | -3.68001 | 0.000233 | rs429358 | All | ApoB | CGPS | 40 ~ 70 |
| -0.00078 | 0.000327 | -2.37469 | 0.017568 | rs429358 | Women | ApoB | CGPS | 40 ~ 70 |
| -0.00138 | 0.000432 | -3.19048 | 0.001422 | rs429358 | Men | ApoB | CGPS | 40 ~ 70 |
| 0.002067 | 0.000902 | 2.292281 | 0.021892 | rs11591147 | All | ApoB | CGPS | 40 ~ 70 |
| 0.001987 | 0.001108 | 1.793538 | 0.072895 | rs11591147 | Women | ApoB | CGPS | 40 ~ 70 |
| 0.002298 | 0.001457 | 1.577982 | 0.11458 | rs11591147 | Men | ApoB | CGPS | 40 ~ 70 |
| -0.00206 | 0.000434 | -4.7515 | 2.02e-06 | rs429358 | All | TG | CGPS | 40 ~ 70 |
| -0.00182 | 0.000547 | -3.33016 | 0.000869 | rs429358 | Women | TG | CGPS | 40 ~ 70 |
| -0.00256 | 0.000691 | -3.70786 | 0.000209 | rs429358 | Men | TG | CGPS | 40 ~ 70 |

Abbreviations: ApoB, apolipoprotein B; SNP, single-nucleotide polymorphisms; TG, triglyceride. Study: EstBB, Estonian Biobank; CGPS, Copenhagen General Population Study.

**Figure S1. Associations between the genotypes of the lead SNPs and the corresponding phenotypes for age-stratified analyses in women and men from UK Biobank.** Abbreviations: ApoB, apolipoprotein B; EA, effect allele; SNP: single nucleotide polymorphism; TG: triglyceride.
